# Development and Validation of a Stability-Indicating RP-HPLC Method for the Quantitative Analysis of Anagrelide Hydrochloride

**DOI:** 10.3797/scipharm.1112-22

**Published:** 2012-04-16

**Authors:** Sudhakar S. Pujeri, Addagadde M. A. Khader, Jaldappagari Seetharamappa

**Affiliations:** 1 Department of Chemistry, Mangalore University, Mangalagangotri, India.; 2 Department of Chemistry, Karnatak University, Dharwad, India.

**Keywords:** Anagrelide, Stability-indicating, Reverse phase, Validation, Forced degradation

## Abstract

A simple, rapid, and stability-indicating reverse-phase liquid chromatographic assay method was developed for Anagrelide Hydrochloride (ANG) in the presence of its degradation products generated from forced decomposition studies. The HPLC separation was achieved on a C18 Inertsil column (250 mm × 4.6 mm i.d. particle size is 5 μm), using solution A, a mixture of 0.03 M potassium di-hydrogen phosphate pH-adjusted to 3.0 using ortho-phosphoric acid (buffer): methanol: acetonitrile (90:5:5, v/v/v), and solution B, which contains a mixture of buffer: acetonitrile (10:90, v/v). The UV detector was operated at 251 nm while column temperature was maintained at 40°C, and the gradient program had the flow rate of 1.0 mL min^−1^. The developed method was validated as per ICH guidelines with respect to specificity, linearity, precision, accuracy, robustness, and limit of quantification. The method was found to be simple, specific, precise, accurate, and reproducible. Selectivity was validated by subjecting the stock solution of ANG to acidic, basic, photolysis, oxidative, and thermal degradation. The calibration curve was found to be linear in the concentration range of 0.05–152 μg mL^−1^ (R^2^ = 0.9991). The peaks of degradation products did not interfere with that of pure ANG. The utility of the developed method was examined by analyzing the tablets containing ANG.

## Introduction

**Fig. 1:**
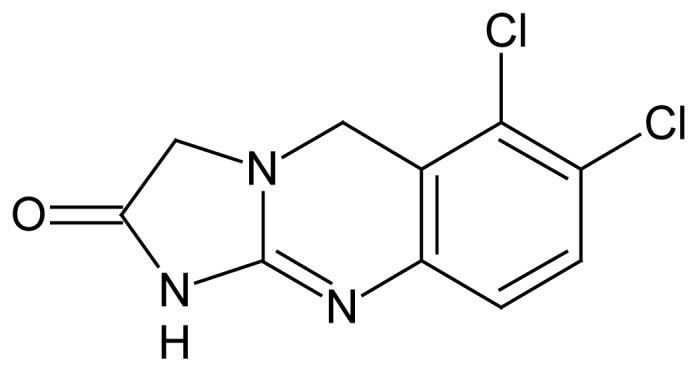
Structure of Anagrelide Hydrochloride

Anagrelide (6,7-dichloro-1,5-dihydroimidazo[2,1-*b*]quinazolin-2(3*H*)-one, fig. 1), is a potent blood platelet reducing agent. Anagrelide (ANG) is a drug used for the treatment of essential thrombocytosis [1]. It works by inhibiting the maturation of megakaryocytes into platelets [2]. Anagrelide hydrochloride was approved by the FDA in 1997 for the treatment of patients with thrombocythemia, secondary to myeloproliferative disorders, to reduce the elevated platelet count and the risk of thrombosis and to ameliorate associated symptoms including thrombo-hemorrhagic events. At therapeutic doses, ANG does not produce significant changes in white cell counts or coagulation parameters, and may have a small but clinically insignificant effect on red cell parameters. ANG inhibits cyclic AMP phosphodiesterase III (PDEIII). PDEIII inhibitors can also inhibit platelet aggregation. However, significant inhibition of platelet aggregation is observed only at doses of ANG higher than those required to reduce platelet count [3, 4].

To the best of the authors’ knowledge, there are only two papers, published in 1987 and 2005, which described the determination of ANG in plasma by GC-MS [5] and LC-MS [6], respectively. A literature search revealed there was no report of validated stability-indicating HPLC method for quantification of ANG in bulk and pharmaceutical formulation. A method used for analysis was validated in accordance with ICH guidelines [7–9]. The present paper describes for the first time the quantitative determination of ANG in bulk samples and formulations. The drug was subjected to stress degradation conditions *viz.*, acidic, basic, oxidation, photolysis and thermal degradation.

## Experimental

### Chemicals and reagents

Pure ANG and its formulation AGRYLIN® was a kind gift from Cipla Ltd, India. HPLC grade acetonitrile and methanol were purchased from Spectrochem, India. Potassium di-hydrogen phosphate, hydrochloric acid, sodium hydroxide and hydrogen peroxide were obtained from Merck (Darmstadt, Germany). HPLC grade water obtained from a Milli-Q water purification system (Millipore, MA, USA) was used throughout the study.

### Instrumentation

All HPLC measurements were made on a Waters 2695 separation module equipped with photo diode array detector 2996 module with data processing on Empower 2.0 version software. pH measurements were made on a pre-calibrated seven multi pH meter (Mettler Toledo Schweraenbach, Switzerland). Mobile phase and sample/standard preparation were degassed by using sonicator (S.V.Scientific, India) and for the filtration of formulation solutions, nylon-66 membrane syringe filter (Nupore, Ghaziabad, India) were used.

### Chromatographic Conditions

The chromatographic column used was Inertsil C18, 250 mm × 4.6 mm i.d. with particle size of 5 μm. The gradient LC method employs solution A and B as mobile phase. The solution A contains a mixture of 0.03 M potassium di-hydrogen phosphate pH adjusted to 3.0 using orthophosphoric acid (buffer): methanol: acetonitrile (90:5:5, v/v/v) and solution B contains a mixture of buffer: acetonitrile (10:90, v/v). The flow rate of the mobile phase was 1.0 mL min^−1^. The HPLC program was set as time (time)/%solution B: 0/30, 1/30, 15/70, 25/70, 30/30, 35/30 with a post run time of 5 min. The column temperature was maintained at 40°C and the detection was monitored at a wavelength of 251 nm. The injection volume was 10 μL. a mixture of water: methanol: acetonitrile (25:50:25, v/v/v) was used as diluent. Both mobile phase and diluent were filtered through a 0.45 μm filter paper (Millipore, Bedford, USA).

### Preparation of Standard Solutions

A stock solution of ANG (1.0 mg mL^−1^) was prepared by dissolving appropriate amount in the diluent. A standard solution of the drug was prepared by suitable dilution.

### Analysis of Pharmaceutical Formulation

Ten tablets of ANG were finely powdered. An amount equivalent to 10 mg of the drug was weighed accurately and transferred into a 100 mL beaker. Using a mechanical stirrer, the powder completely disintegrated in the methanol. The solution was filtered through nylon-66 membrane syringe filter (Nupore, Ghaziabad, India) and 2 mL of methanol was used for two times to rinse the syringe filter and then filtrate was made up to 10 mL with the methanol. It was further diluted with diluent for analysis.

### Validation

Validation studies were conducted using the optimized assay conditions based on the principles of validation described in the ICH guidelines. Key analytical parameters including specificity, accuracy, precision, linearity, detection limit and quantitation limit were evaluated.

#### Specificity and Selectivity

Specificity is the ability of the method to measure the analyte response in the presence of its potential impurities or degradation products. The presence or absence of peaks due to excipients, impurities and degraded products was examined to evaluate the assay of the drug substance. Stress testing of the drug substance can in turn help establish the degradation pathways and the intrinsic stability of the molecule and validate the stability-indicating power of the analytical procedures used.

The specificity of the developed LC method for ANG was determined in the presence of its impurities. Forced degradation studies were also performed on ANG to provide an indication of the stability-indicating property and specificity of the proposed method. The stress conditions employed for degradation study include light (carried out as per ICH QB), heat (80°C), acid hydrolysis (1M HCl), base hydrolysis (0.1M NaOH) and oxidation (3% H_2_O_2_). For heat and light studies, study period was 10 days whereas for acid, base hydrolysis and oxidation, it was 48 h.

#### Precision

Assay method precision was evaluated by carrying out six independent assays of test sample of ANG against qualified reference standard. The percentage of R.S.D of six assay values obtained was calculated. The intermediate precision of the method was also evaluated using different analyst and a different instrument in the same laboratory.

#### LOD and LOQ

The LOD and LOQ were determined at a signal to noise ratio of 3:1 and 10:1, respectively, by injecting a series of dilute test solutions of known concentrations. Precision study was also carried out at the LOQ level by injecting six preparations and calculating the percentage of R.S.D of the area.

#### Linearity and Range

Linearity test solutions for assay method were prepared from stock solution at six concentration levels from 25 to 150% of assay analyte concentration. The peak area versus concentration data was performed by least-squares linear regression analysis. The linearity test was performed for 3 consecutive days in the same concentration range for assay method. The percentage of R.S.D value of the slope and y-intercept of the calibration curve was calculated. Linearity solutions containing the drug in the range of 0.05–152 μg mL^−1^ were used. The solutions were injected in triplicate into the HPLC column keeping the injection volume constant (10μL) and chromatograms were recorded.

#### Accuracy

The accuracy of the assay method was evaluated in triplicate at different concentration levels, i.e 50, 100 and 150μg mL^−1^ in bulk drug sample. The percentage of recoveries was calculated from the slope and y-intercept of the calibration curve obtained. Accuracy/recovery experiments were performed in triplicate. It was also evaluated by fortifying a mixture of formulation sample with three known concentrations of the drug. The recovery of the added drug was determined and in turn % of recovery was calculated.

#### Robustness

The purpose of robustness test is to check whether the chromatographic performance is affected by small changes in operating conditions, as well as to provide evidence that given small modifications of the system still meet the system suitability requirements. To determine the robustness of the developed method, experimental conditions were purposely altered to check the reproducibility, resolution between ANG and nearest impurity and quantitative recovery of the drug. The flow rate of the mobile phase was 1.0 mL min^−1^. To study the effect of flow rate on the resolution, it was changed by 0.2 units from 0.8 to 1.2 mL min^−1^. The effect of pH on resolution of impurities was studied by varying ± 0.1 pH units. The effect of column temperature on resolution was studied at 35°C and 45°C instead of 40°C. In the aforementioned varied conditions, the components of the mobile phase were held constant as stated. Chromatograms were recorded and compared with the optimum chromatographic conditions mentioned earlier.

#### Solution Stability and Mobile Phase Stability

The solution stability of ANG in the assay method was carried out by leaving both the test solutions of the sample and reference standard in tightly capped volumetric flasks separately, at room temperature, up to the study period of 48 h. The chromatograms of these solutions were recorded separately with an interval of 1 h up to 48 h and the peak responses were compared.

The mobile phase stability was carried out by assaying the freshly prepared bulk drug and formulation sample solutions against freshly prepared reference standard solution with an interval of 8 h. The same mobile phase was used throughout the experiment. %RSD values were calculated for mobile phase and solution stability experiments.

### Forced Degradation Studies

The forced degradation studies were performed to evaluate the stability of the ANG method. The study was performed by using the conditions of dry heat, hydrolysis, oxidation and photolysis, as per ICH guidelines. Under a particular stress condition, the drug molecule may generate different degradation products. The degradation products generated in the stressed samples are generally termed as “potential” degradation products which might be formed under relevant storage conditions. The hydrolytic degradation of a newly proposed drug in acidic and alkaline conditions can be studied by refluxing the same in HCl/NaOH. Furthermore, to examine the degradation by oxidation, it is suggested to use hydrogen peroxide in the concentration range of 3–30%. The degradation studies carried out in the present work are described below.

#### Oxidation of ANG

For oxidation study, 10.0 mL of stock solution of ANG (100 μg mL^−1^) was transferred to 20 mL amber colored volumetric flask and the volume was made up to 20 mL with 10% hydrogen peroxide. The flask was placed at 80°C for 48 h, cooled to room temperature and the volume was readjusted with 3% hydrogen peroxide. Then the solution was filtered through a 0.45 μm syringe filter and 10 μL was injected into the liquid chromatographic system to detect peak of degradents of the oxidation.

#### Thermal Degradation of ANG

This was carried out by transferring 10.0 mL of ANG (100 μg mL^−1^) into a 20 mL volumetric flask and diluting to the mark with the mobile phase. The flask was closed and placed at 80°C for 10 days, cooled to room temperature and volume of solution was readjusted with diluent. Chromatogram was recorded.

#### Degradation of ANG by Acid

For this, 10.0 mL of ANG (100 μg mL^−1^) was transferred into a 20 mL volumetric flask and the volume was made up to the mark with 1 M hydrochloric acid. The flask was placed at 80°C for 48 h, cooled to room temperature and the volume of solution was readjusted to 20 mL with 0.1 M hydrochloric acid. The solution was adjusted to neutral pH by using 1 M sodium hydroxide, to avoid column damage at too acidic of conditions, and it leads to peak splitting or unsymmetrical peak shape. The neutral solution was injected to liquid chromatograph and chromatogram was recorded.

#### Degradation of ANG by Alkali

This was conducted by transferring 10.0 mL ANG (100 μg mL^−1^) in-to a 20.0 mL volumetric flask and diluting to the mark with 0.1 M sodium hydroxide. The flask was kept at 80°C for 48 h, cooled to room temperature and volume was readjusted to 20 mL with 0.1 M sodium hydroxide. The solution was neutralized with 0.1 M hydrochloric acid solution and then chromatogram was recorded.

#### Photo Degradation of ANG

For photo degradation 10.0 mL of ANG (100 μg mL^−1^) was transferred into a 20.0 mL volumetric flask and diluted to the mark with mobile phase. The flask was exposed to UV light for 10 days continuously. The experiment was also repeated with solid drug sample. The solutions of both were injected into liquid chromatograph separately and chromatograms were recorded.

## Result and Discussion

### Method Development and Optimization

To optimize the chromatographic conditions, the effect of composition of mobile phase, the flow rate and the detection wavelength were investigated. An appropriate chromatographic condition was particularly required in this method for the degradation products and drug substance. Firstly, different chromatographic columns were tested including ODS Hypersil, Zorbax C18, Zorbax Eclipsed XDB C8, waters XTerra RP18, Thermo BDS, Waters symmetry shield RP18 and Inertsil ODS columns. There were two pairs of degradation product peaks that had close retention times in the chromatogram of crude drug substance and also in the forcibly degraded drug substance. Moreover, the peaks of degradents and of the pure drug were not separated. Only the utilization of inertsil ODS-3 column could simultaneously obtain the baseline separation. Secondly, the composition of mobile phase was investigated. The mixture of methanol and acetonitrile had better peak resolution than acetonitrile alone. Orthophosphoric acid was better than formic acid and Trifluroacetic acid (TFA) in peak separation. It was also found that the concentration of potassium di-hydrogen phosphate was crucial for the baseline separation of the aforementioned two pairs of peaks and the 0.03M strength was screened as the best concentration. Finally, the monitoring wavelength was set at 251 nm, which is the characteristic and maximum absorption of drug substance according to their 3D ultraviolet absorption spectra. The relative higher column temperature (40°C) and lower flow rate (1.0 mL min^−1^) were employed to obtain better resolution based on the analysis of different conditions. System suitability parameters obtained for ANG are shown in Table 1.

**Fig. 2:**
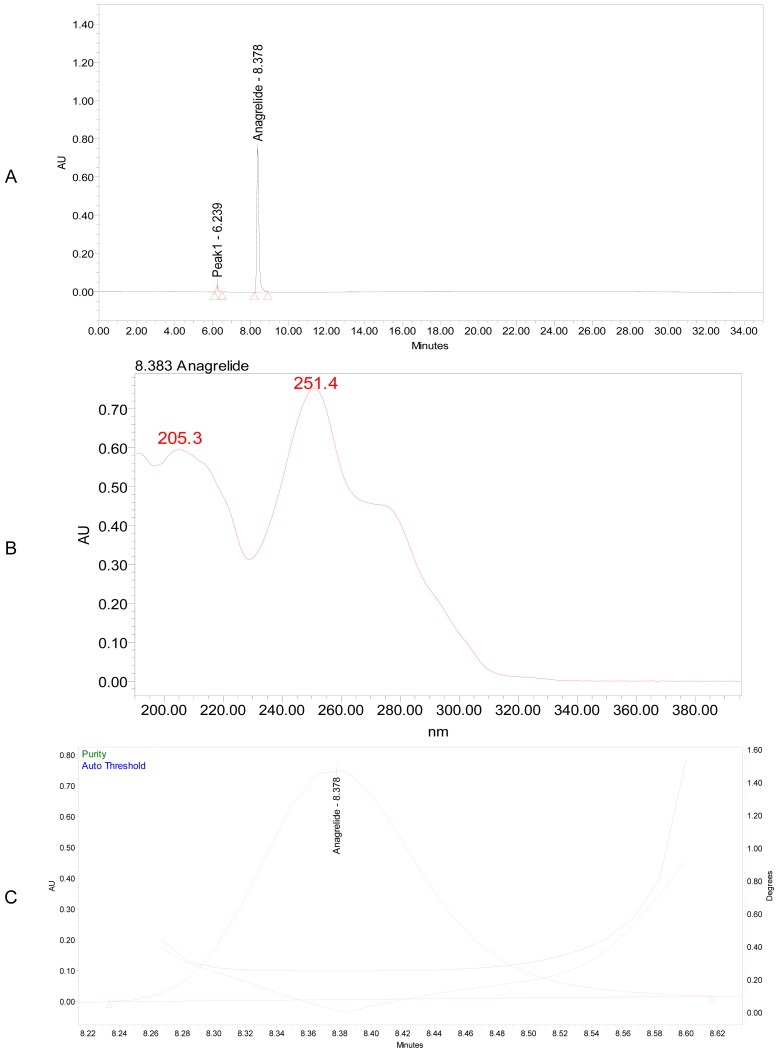
A typical chromatogram (a), UV spectrum (b) and purity plot (c) of ANG

Well-resolved and symmetric peaks were noticed with the solution A, which contains a mixture of 0.03 M potassium di-hydrogen phosphate pH adjusted to 3.0 using ortho-phosphoric acid (buffer): methanol: acetonitrile (90:5:5, v/v/v), while solution B contains a mixture of buffer: acetonitrile (10:90, v/v). The flow rate of the mobile phase was 1.0 mL min^−1^ and the detection wavelength was 251 nm. Typical chromatogram, purity plot and UV absorbance spectra are shown in Fig. 2.

### Results of Validation Studies

#### Precision

The RSD values for intra-day and inter-day precision study were 1.1 % and 1.5 %, respectively. This indicates that the proposed method was sufficiently precise.

#### Accuracy

The % recovery values of ANG in pharmaceutical formulation ranged from 99.4 to 100.3. High percentage recovery values revealed that the proposed method is accurate and could be adopted for routine quality control.

#### Limit of Detection (LOD) and Limit of Quantification (LOQ)

The ICH guidelines were followed to calculate the values of LOD and LOQ and were observed to be 0.019 μg mL^−1^ and 0.054 μg mL^−1^, respectively, and obtained %RSD at LOQ level was 1.2.

#### Linearity

The calibration plot for the assay of ANG was linear over the concentration range of 0.05–152 μg mL^−1^ (R^2^=0.9991).

#### Robustness

Under all the deliberately varied chromatographic conditions (flow rate, pH and column temperature), the reproducibility of results was observed to be reasonably good. In all the deliberately varied parameters the resolution between drug substance and degradation products was >2.0 min. Hence, the proposed method has good robustness for the assay of ANG in bulk or in tablets.

#### Solution Stability and Mobile Phase Stability

The % RSD values for the assay of ANG during solution stability and mobile phase stability experiments were found to be less than 0.9 %. This indicated that the sample solutions and mobile phases used during the assay were stable for at least 48 h.

### Results of Forced Degradation Studies

**Fig. 3:**
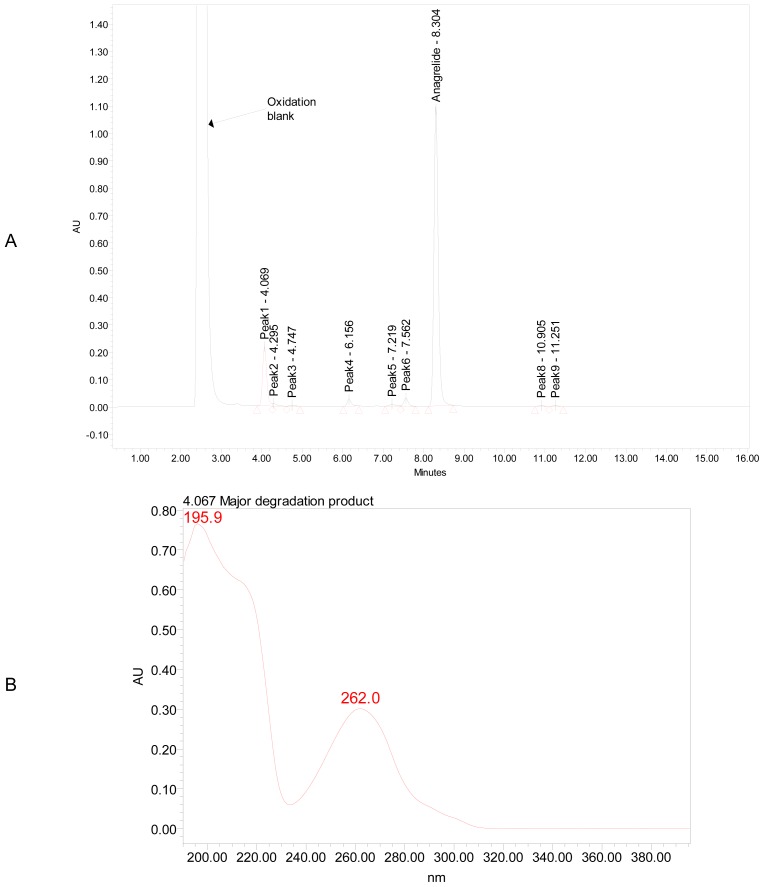
A typical chromatogram (a) and major degradation product (RT=4.069 min) UV spectrum (b) of ANG exposed to 3 % hydrogen peroxide

**Fig. 4:**
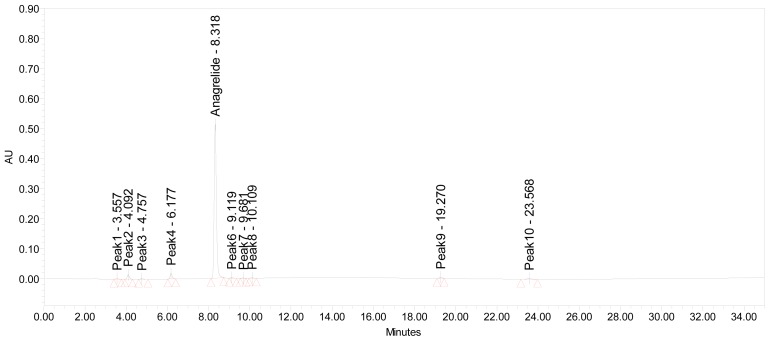
A typical chromatogram of ANG exposed to 80°C (Thermal)

**Fig. 5:**
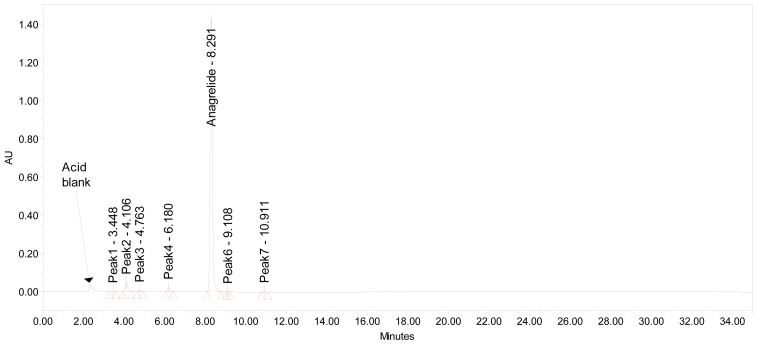
A typical chromatogram of ANG exposed to 0.1 M hydrochloric acid

**Fig. 6:**
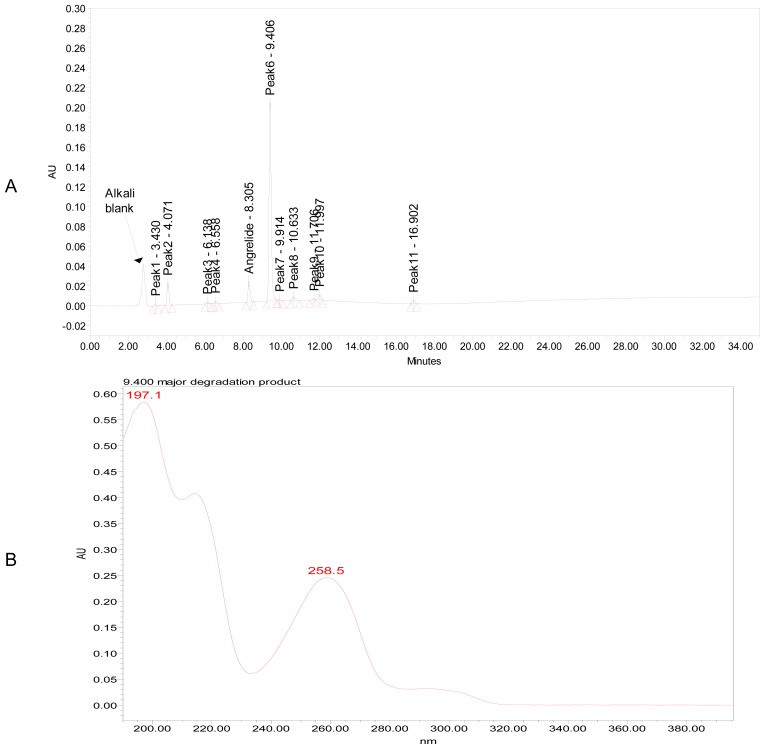
A typical chromatogram (a) and major degradation product (RT=9.406 min) UV spectrum (b) of ANG exposed to 1 M sodium hydroxide

Significant degradation was observed in oxidation of ANG sample (Fig. 3), 15% major degradation product was observed at retention time of 4.069 min and having maximum absorbance at 195.9 and 262 nm. There was no significant degradation of ANG upon exposure to dry heat at 80°C for 10 days (fig. 4), and the total impurities increased from 2.9 to 3.2 area percentages. In acid hydrolysis (fig. 5), no significant change was observed, which indicated that the drug was stable against these stress conditions. Similar stable behavior was observed upon exposure of solid drug to the combined conditions of light (UV and Visible light), figures not shown. However, the exposure of drug to alkali hydrolysis resulted in major degradation (fig. 6), yielding four products resolving at relative retention time (RRT) of 0.49, 1.13, 1.28 and 1.44. The area percentage of major degradation product observed at RRT 1.13 is 76.12 and it has maximum absorption at 197.1 and 258.5 nm. Peak purity test results confirmed that the ANG peak was homogenous and pure in all the analyzed stress samples. Lower purity angle value of ANG compared to that of the purity threshold revealed that the ANG was free from interference from its impurities and its degradents.

To avoid column damage at too acidic or too basic conditions generated during the stress studies and also to avoid peak splitting or unsymmetrical peak, the solutions were neutralized and chromatograms were recorded. The peak purity of the drug substance was checked using a PDA detector. The purity angle was within the purity threshold limit obtained in all stressed samples and demonstrated the analyte peak homogeneity. Recovery results are shown in Table 2. Assay of the stressed samples was performed by comparison with reference standards and the mass balance (% assay + % degradation products) for stressed samples was calculated (Table 3). The developed HPLC method revels that there is no interference from the impurities, degradation products and formulation ingredients to determine the assay of the drug substance in pharmaceutical formulation and in bulk drugs.

### Analysis of Pharmaceutical Preparations

The proposed method was successfully applied to the analysis of ANG drug substance in formulation and the results are shown in Table 4. The low values of relative standard deviation indicated high precision of the method. High percent recovery values indicated that the commonly employed excipients Anhydrous Lactose NF, Crospovidone NF, Lactose Monohydrate NF, Magnesium stearate NF, Microcrystalline cellulose NF, Povidone USP., did not interfere in the analysis of ANG in capsules.

## Conclusions

In this study, it was possible to develop a selective and validated stability-indicating HPLC assay method for Anagrelide hydrochloride on an ODS-3 column, which could separate the drug and its degradation products formed under a variety of stress conditions. The described method was found to be rugged, linear, reproducible, specific, accurate and capable of separating drug substance from its degradation impurities. Anagrelide can be determined in bulk powder, pharmaceutical formulation as well as in the presence of its degradation products by HPLC method. ICH guidelines were followed throughout the study for method validation and stress testing. In view of this, the proposed method could be adopted for quality control and routine analysis.

## Figures and Tables

**Tab. 1:** System suitability parameters

Retention Time	Purity Angle	Purity Threshold	USP Tailing	USP Plate Count
8.378	0.099	0.256	1.198	29880

**Tab. 2:** Recovery data for ANG spiked into a mixture of stressed samples.

Spiked Concentration (μg mL^−1^)^a^	Calculated Concentration (μg mL^−1^ ± SD., R.S.D (%)	Recovery (%)
50.0	49.7 ± 0.07., 0.95	99.4
100.0	100.2 ± 0.09., 0.27	100.2
150.0	150.5 ± 0.01., 1.22	100.3

a Average of six determinations.

**Tab. 3:** Summary of forced degradation results

Stress condition	Time (h)	%Assay of ANG	%Mass balance (assay + DP^a^)
Acid hydrolysis (0.1 M HCl), reflux at 80 °C	48	94.38	97.16
Base hydrolysis (1 M NaOH), reflux at 80 °C	48	7.39	97.07
Oxidation (10% H_2_O_2_), reflux at 80 °C	48	78.84	97.10
Thermal (80 °C)	10 days	90.74	97.12
Photolysis (254 nm)	10 days	95.01	97.17

a Degradation products.

**Tab. 4:** Analysis of ANG in pharmaceutical formulations.

Formulation	Labeled, mg	Found^a^, mg	% RSD	% Recovery
AGRYLIN®	0.5	0.496	0.69	99.2

a Average of nine determinations.
